# Patterns of introgression vary within an avian hybrid zone

**DOI:** 10.1186/s12862-021-01749-1

**Published:** 2021-01-28

**Authors:** Logan M. Maxwell, Jennifer Walsh, Brian J. Olsen, Adrienne I. Kovach

**Affiliations:** 1 Department of Natural Resources and the Environment, University of New Hampshire, Durham, NH USA; 2 Fuller Evolutionary Biology Program, Cornell Laboratory of Ornithology, Ithaca, NY 14850 USA; 3 School of Biology & Ecology, University of Maine, Orono, ME USA

**Keywords:** *Ammospiza caudacutus*, *Ammospiza nelsoni*, Hybrid zone, Introgression, Natural selection, Sexual selection, Demographics, Haldane’s rule, Assortative mating, Exogenous selection

## Abstract

**Background:**

Exploring hybrid zone dynamics at different spatial scales allows for better understanding of local factors that influence hybrid zone structure. In this study, we tested hypotheses about drivers of introgression at two spatial scales within the Saltmarsh Sparrow (*Ammospiza caudacuta*) and Nelson’s Sparrow (*A. nelsoni*) hybrid zone. Specifically, we evaluated the influence of neutral demographic processes (relative species abundance), natural selection (exogenous environmental factors and genetic incompatibilities), and sexual selection (assortative mating) in this mosaic hybrid zone. By intensively sampling adults (n = 218) and chicks (n = 326) at two geographically proximate locations in the center of the hybrid zone, we determined patterns of introgression on a fine scale across sites of differing habitat. We made broadscale comparisons of patterns from the center with those of prior studies in the southern edge of the hybrid zone.

**Results:**

A panel of fixed SNPs (135) identified from ddRAD sequencing was used to calculate a hybrid index and determine genotypic composition/admixture level of the populations. Another panel of polymorphic SNPs (589) was used to assign paternity and reconstruct mating pairs to test for sexual selection. On a broad-scale, patterns of introgression were not explained by random mating within marshes. We found high rates of back-crossing and similarly low rates of recent-generation (F1/F2) hybrids in the center and south of the zone. Offspring genotypic proportions did not meet those expected from random mating within the parental genotypic distribution. Additionally, we observed half as many F1/F2 hybrid female adults than nestlings, while respective male groups showed no difference, in support of Haldane’s Rule. The observed proportion of interspecific mating was lower than expected when accounting for mate availability, indicating assortative mating was limiting widespread hybridization. On a fine spatial scale, we found variation in the relative influence of neutral and selective forces between inland and coastal habitats, with the smaller, inland marsh influenced primarily by neutral demographic processes, and the expansive, coastal marsh experiencing higher selective pressures in the form of natural (exogenous and endogenous) and sexual selection.

**Conclusions:**

Multiple drivers of introgression, including neutral and selective pressures (exogenous, endogenous, and sexual selection), are structuring this hybrid zone, and their relative influence is site and context-dependent.

## Background

Understanding hybrid zone structure, including spatial patterns of introgression and character variation, can help infer processes that maintain hybrid zones and provide important insights into the nature of species boundaries [1–3]. Spatial variation in hybridization rates and outcomes can reveal correlations between hybrid-zone structure and specific features of local environmental conditions as well as local population dynamics [4]. Outcomes of hybridization and introgression can vary based on numerous factors, including local population demographics (relative species abundances), exogenous natural selection (environmental effects on hybrid fitness), endogenous natural selection (genetic incompatibilities and heterosis), and sexual selection (mate competition and mating preferences). Identifying the relative influence of these processes in a hybrid zone is important for understanding spatial variation in introgression and predicting future hybrid zone dynamics.

Differences in rates of hybridization and patterns of introgression due to local demographics and population size have been seen in a variety of taxa, including birds [5–8], and can play a key role in hybrid zone structure. Hubbs Principle [9] asserts that if population sizes are unequal between parental species, hybridization will be more widespread due to restricted mate choice for the rarer species [10]. Further, when one species is rare relative to the other, hybrid fertilizations may constitute a larger proportion of the total matings of the rarer species, and if hybrids backcross differentially to the common parental taxa, this can lead to genetic assimilation [6, 11]. For example, in the Golden-winged (*Vermivora chrysoptera*)- Blue-winged Warbler (*V. pinus*) hybrid zone, rates of introgression vary across sites that differ in relative population size and status of the two species, such that when Golden-winged Warbler populations were at a minimum, introgression was more prevalent than in populations with more equal proportions of the two species [7]. However, if parental populations are highly skewed toward one species, the absolute rate of hybridization may be limited due to the reduced number of individual interactions between the two species. This can be especially true in promiscuous mating systems that depend on encounter rates, such that members of the rarer species may be less likely to find mates [12].

Patterns of introgression can also vary substantially across hybrid zones due to variation in hybrid offspring or parental species fitness in the local environment. Differential adaptation along environmental gradients explain patterns of hybridization and introgression in many systems [5, 13–15]. In particular, habitat preference plays a critical role in the fine-scale structure of mosaic hybrid zones [14, 16]. In plants, for example, variable rates of hybridization and reproductive isolation have been attributed to both local variation in pollinator behavior [17] and elevational differences [18]. In animal systems, complex mosaics of parental and hybrid genotypes can occur in environments that are intermediate between parental habitat characteristics [16] where environmental gradients predict patterns of introgression [13].

Endogenous selective factors may also play a part in hybrid zone structure, whereby adaptation is on the genomic level and independent of the external environment [19, 20]. Hybrid individuals may be selected against due to genetic incompatibilities leading to inviability/sterility or reduced fitness, and this in turn can contribute to maintaining species boundaries [21, 22]. Selection against hybrids may also differ between sexes. For example, Haldane’s Rule predicts that the heterogametic sex of first-generation hybrids should experience greater reductions in fitness [23, 24]. This reduced fitness can result from lower fertility or survival, both of which have been observed in avian hybrid zones [20, 24].

Interspecific competition and assortative mating have also been shown to influence patterns of hybridization and introgression across hybrid zones by means of sexual selection. Some mating behaviors may promote hybridization and gene flow, while others may inhibit it. One species may have a consistent competitive mating advantage or dominance over the other, regardless of the species identity of the mate, resulting in unidirectional gene flow and asymmetrical introgression [25, 26]. Alternatively, assortative mating may preserve species boundaries and maintain bimodal population structure due to pre- or post-copulatory processes [27].

**Fig. 1:**
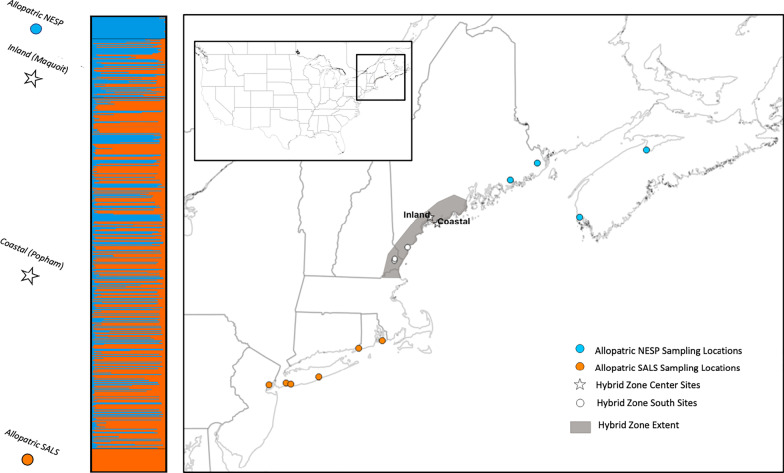
Map depicting Saltmarsh and Nelson’s Sparrow sampling locations and admixture plot from STRUCTURE. The map on the right has orange dots as allopatric Saltmarsh Sparrow sampling locations, while blue dots are allopatric Nelson’s Sparrow sampling locations. White stars on the map indicate coastal and inland sites where this study was conducted, while white dots indicate previous study locations (not included in the admixture plot) that provided broad scale comparisons to our two sites as representative of the southern hybrid zone for this study. The hybrid zone extent is shown shaded grey on the map. The admixture plot on the left depicts the central inland and coastal study locations and allopatric populations, where each vertical bar represents the genetic makeup of an individual, blue representing the Nelson’s Sparrow alleles, and orange representing the Saltmarsh Sparrow alleles. Sparrows of pure ancestry have a bar of a solid color, while sparrows of mixed ancestry have bars comprised of both colors

In this study, we investigated patterns of introgression and drivers of gene flow at broad and fine spatial scales in the Saltmarsh Sparrow (*Ammospiza caudacuta*) and Nelson’s Sparrow (*A. nelsoni*) hybrid zone. These two sparrow species have restricted breeding habitat along the northeastern Atlantic coast of the United States. Nelson’s Sparrows breed in marshes from the Canadian Maritimes to Massachusetts and the Saltmarsh Sparrow’s range extends from southern Maine to Virginia [28, 29]. These sister species co-inhabit marshes where their ranges overlap, forming a ~ 200 km, linear hybrid zone along the New England coast ([30–34]; Fig. 1). Saltmarsh Sparrows are entirely restricted to tidal salt marshes, while Nelson’s Sparrows will also breed in brackish, less tidal coastal marshes, and have been known to inhabit hayfields and fens [28, 31, 35]. In turn, Saltmarsh Sparrows show a number of adaptations that increase their fitness in fully estuarine marshes relative to Nelson’s Sparrows [36, 37].

The wetland habitats within the Saltmarsh-Nelson’s Sparrow hybrid zone are patchy, with a complex spatial structuring of marsh types [38]. Previous work indicates that high levels of introgression exist across the zone; however, levels of admixture vary spatially in accordance with a mosaic hybrid zone model, and species boundaries remain largely intact in the face of high gene flow [39, 40]. Studies in the southern edge of the hybrid zone identified asymmetrical introgression towards the Saltmarsh Sparrow, reduced occurrence of female hybrids relative to males, and assortative mating [32, 40, 41]. Relative species densities in these populations, however, are highly skewed (Saltmarsh to Nelson 5.5:1) and very few intermediate (F1/F2) individuals exist in that area [41]. It is unknown, therefore, whether patterns of introgression are driven by the adaptive benefits of Saltmarsh Sparrow alleles in just these marshes or across the hybrid zone, by selection against hybrids, by mate preference or competition, or simply by random mating in the face of skewed species distributions. Comparing patterns of introgression across spatial locations with differing habitats, intrapopulation densities, and relative species distributions will yield insight into potential drivers of the structure and maintenance of this hybrid zone.

Here we tested four hypothesized drivers of introgression within the Saltmarsh-Nelson’s Sparrow hybrid zone—(1) neutral demographic processes, (2) exogenous selective forces, (3) endogenous selective forces, and (4) sexual selection. We sought evidence for these drivers by comparing degree and direction of introgression, distribution of adult and offspring genotypes and sex ratios, and mating patterns at two spatial scales—(1) broadly between sites in the center and southern edge of the hybrid zone and (2) at a finer scale in the center of the zone between coastal and inland habitats. We made the following predictions about rates of hybridization and introgression for each hypothesized driver (summarized also in Table 1):

1. Neutral demographic processes—Patterns of introgression are explained by random mating and differ as a function of relative abundance of the two inter-breeding species. Nelson’s sparrows are rare in the southern end of the hybrid zone and less common on coastal than inland sites, whereas the two species occur in more similar numbers in the center of the zone and on inland sites. As a result, we would expect higher rates of introgression, a greater number of recent-generation hybrids, and more symmetrical back-crossing in the center vs. southern portions of the hybrid zone and in inland vs. coastal sites.
2. Exogenous selective forces—Patterns of introgression will be driven by exogenous selection as a result of environmental variation in parental and hybrid fitness with respect to coastal and inland sites. Because males do not provide any parental care, we expect that exogenous selective pressures related to nesting success would act more strongly on females than males, resulting in more Saltmarsh Sparrow females on the coastal site and more Nelson’s Sparrow females on the inland site, consistent with the species’ habitat affinities. Further, at the inland site, we expect Saltmarsh Sparrows to hybridize more than expected by chance and for hybrids to backcross more toward Nelson’s Sparrow than expected by mate availability. Likewise, Nelson’s Sparrows should hybridize more and there should be more backcrossing toward Saltmarsh Sparrows at the coastal site than is expected by random mating.
3. Endogenous selective forces—Patterns of introgression will be shaped by endogenous selective factors, reflecting environmentally independent genetic incompatibilities of hybrid genotypes. As predicted by Haldane’s Rule, hybrid females will have reduced fitness, resulting in a deficit of recent-generation hybrid females at all sites. This may manifest during either offspring production (i.e. male-biased offspring sex ratio) or via a reduction in juvenile and/or adult survival of females.
4. Sexual selection—Patterns of introgression will be shaped by sexual selection, such that Saltmarsh and Nelson’s Sparrows will exhibit mating preferences (e.g., avoidance of interspecific mating) or competitive abilities across demographic and environmental contexts. If true, we would expect similar departures from random mating for each species, regardless of local variation in intraspecific mate availability or environment. For instance, if the asymmetrical introgression and assortative mating reported previously in the southern end of the hybrid zone was driven by these processes, we would expect to see similar rates of asymmetry and F1/F2s across the hybrid zone after controlling for local mate availability.

**Table 1:** Four drivers of hybridization and introgression tested within this study and associated prediction for each

Driver	Prediction
Demographic processes (relative population density)	Amount/direction of introgression, as well as distribution of genotypic classes will be directly proportional to the relative abundance and mate availability of the two species, with higher rates and more symmetrical patterns of introgression in the center and inland sites vs. southern and coastal sites
Natural selection (exogenous environmental factors)	Distributions of adult and offspring genotypes, including amount and direction of introgression, will reflect the species’ differential habitat and nesting affinities of Nelson’s Sparrows to inland sites and Saltmarsh Sparrows to coastal sites, regardless of mate availability
Natural selection (endogenous factors)	Selection against hybrid females through genetic incompatibilities will result in male-biased production of hybrid offspring and/or reduced survival of hybrid females from nestling to adult stage
Sexual selection (environmentally independent)	Interspecific mating will be avoided, such that proportion of inter-specific mating events will be less than expected from the availability of interspecific mates

## Results

### Patterns of introgression

**Fig. 2:**
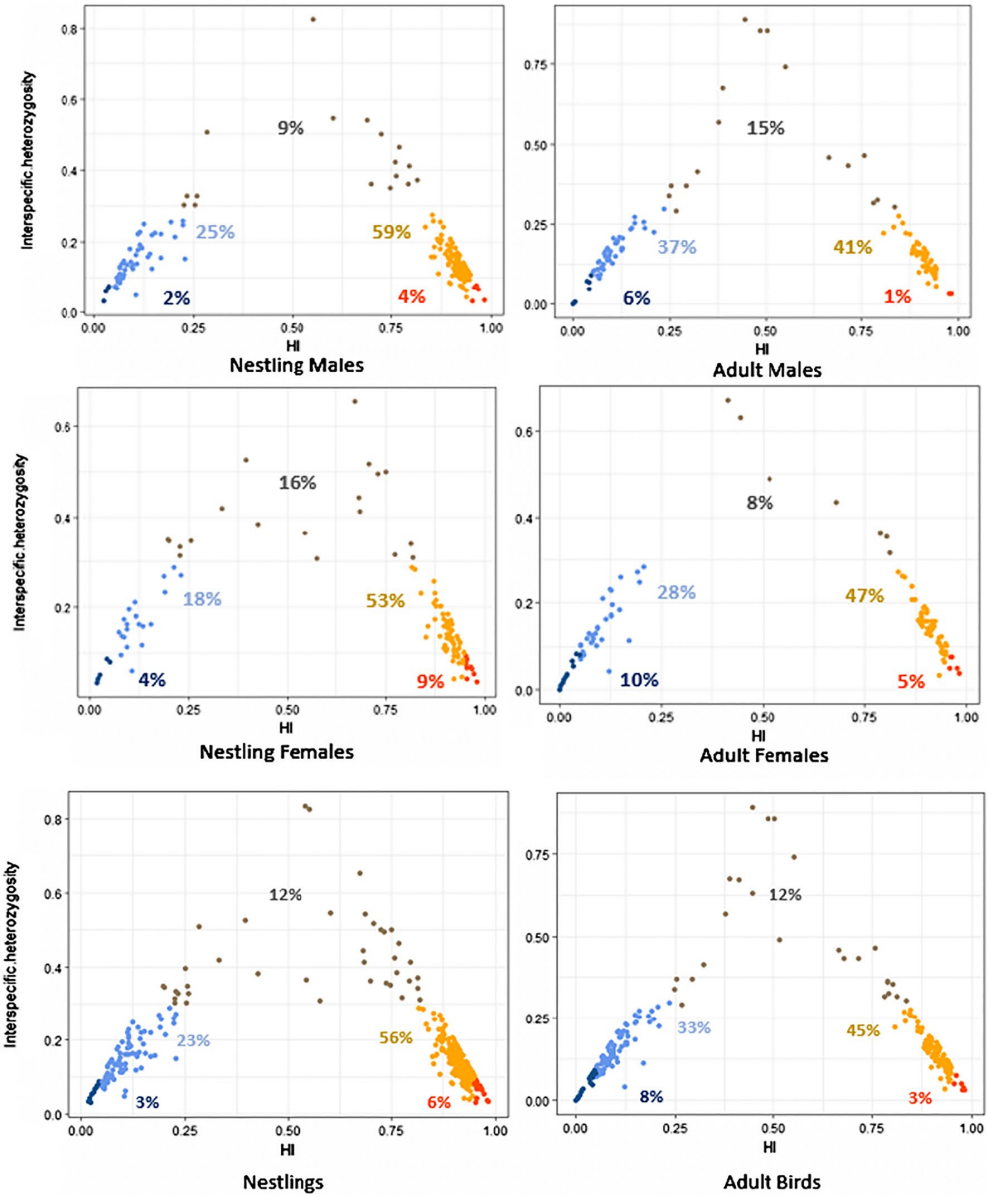
Genetic composition (hybrid index (HI)/interspecific heterozygosity) in the center of the Saltmarsh-Nelson’s hybrid zone. The bottom panel shows the distribution of genetic composition for all nestling and adult birds, and the upper two panels show the distributions by sex. Colored circles indicate the corresponding genotypic class for the combination of HI and interspecific heterozygosity as follows: dark blue = pure Nelson’s Sparrows, light blue = backcrossed Nelson’s Sparrows, gray = recent generation hybrids, yellow = backcrossed Saltmarsh Sparrows, and orange = pure Saltmarsh Sparrows. Percentages represent the frequency of each genotypic class within the respective frame

We banded and genotyped 544 sparrows (218 adults, 326 nestlings and eggs) across the coastal (Popham) and inland (Maquoit) study sites in the center of the hybrid zone over 2 breeding seasons. STRUCTURE analysis revealed extensive backcrossing at each of the two study sites (Fig. 1). Although few individuals exhibited pure ancestry, most shared a larger proportion of alleles from one parental species than the other (i.e., backcrossed). Using a hybrid index to classify individuals into genotypic classes, 33% of adults were backcrossed Nelson’s Sparrows (30 females, 42 males), 45% were backcrossed Saltmarsh Sparrows (50 females, 47 males), 12% were recent generation hybrids (8 female, 17 male), 8% were pure Nelson’s Sparrows (11 females, 7 males), and 3% were pure Saltmarsh Sparrows (5 females, 1 male; Fig. 2; Table 2). The mean hybrid index was similar between adult males (0.54 ± 0.15) and females (0.57 ± 0.16); and was slightly higher for nestlings (0.65 ± 0.13), although still similar between the sexes (Table 3). Interspecific heterozygosity was comparable among adult male (0.12 ± 0.03), adult female (0.15 ± 0.01), and nestling birds (male: 0.17 ± 0.01; female: 0.20 ± 0.01); Table 3). The mean hybrid index was higher among nestlings than adult birds for both males (t = − 3.37, *P*≤ 0.001) and females (t = -2.13, *P* = 0.03; Table 3). The genotypic structure of the population was similar between sampled adults and nestling birds, indicating no reduced survival for any one genotypic class as a whole (Fig. 2). The distribution of genotypic classes in nestlings between the inland and coastal habitats in the center of the hybrid zone illustrates considerable current interspecific gene flow, such that most offspring are of backcrossed origins, with fewer recent-generation hybrids, and even fewer pure individuals (Fig. 2).

**Table 2:** Genotypic composition of birds sampled during 2016 & 2017 breeding seasons

	BC-NESP	BC-SALS	F1/F2	NESP	SALS	Adults	Nestlings	Total birds
Coastal site	32% (53)	49% (82)	10% (16)	5% (9)	4% (6)	166	300	466
Inland site	37% (19)	29% (15)	17% (9)	15% (8)	2% (1)	52	26	78
Total birds	33% (72)	45% (97)	12% (25)	8% (17)	3% (7)	218	326	544

Genotypic classes: pure Saltmarsh Sparrow (SALS), pure Nelson’s Sparrow (NESP), first-generation hybrids (F1/F2), backcrossed Saltmarsh Sparrow (BC-SALS), and backcrossed Nelson’s Sparrow (BC-NESP)

**Table 3:** Average hybrid index (0.00–1.00) and interspecific heterozygosity levels across the 2016 & 2017 breeding seasons

	Coastal site	Inland site	Male adults	Female adults	Male nestlings	Females nestlings
Mean hybrid index	0.64	0.43	0.54	0.57	0.62	0.64
Mean interspecific heterozygosity	0.17	0.21	0.12	0.15	0.17	0.20

### Tests of hypotheses

#### Demographic processes

The observed distribution of genotypic classes and direction of introgression in the center of the hybrid zone differed from that previously found in the southern end of the hybrid zone. In the south, the population consisted of 8.0% backcrossed Nelson’s Sparrows, 49% backcrossed Saltmarsh Sparrows, 12% recent-generation hybrids, 4% pure Nelson’s Sparrows, and 27% pure Saltmarsh Sparrows [41]. This distribution differed significantly from what was observed in the center of the hybrid zone (Χ^2^ = 113.7, *P* ≤ 0.001), such that there were many more backcrossed and pure Saltmarsh Sparrows and many fewer backcrossed Nelson’s Sparrows in the southern range margins than the center, where we observed more equal distribution of backcrossed individuals in both the Nelson and Saltmarsh directions and very few pure individuals.

**Fig. 3:**
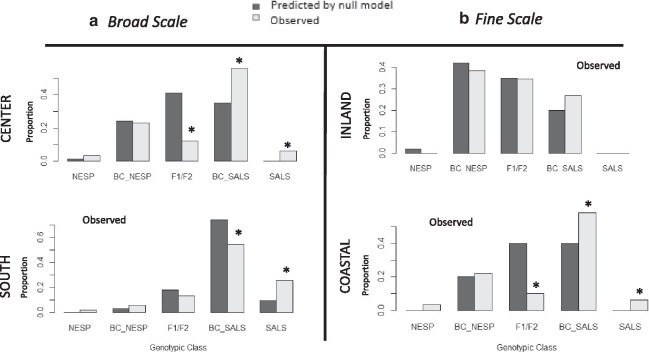
Hypothesis testing for demographic processes model of selection at two spatial scales. The observed distribution of genotypic classes (light gray) and predicted distribution of genotypic classes (dark gray) as predicted from observed relative parental genotypic densities, assuming random mating. Left panel **a** compares patterns at a broad scale in the center and south of the hybrid zone, and right **b** compares fine scale patterns between inland and coastal sites within the center of the hybrid zone. Genotypic classes from left to right: Nelson’s Sparrow (NESP), Backcrossed Nelson’s Sparrow (BC_NESP), recent-generation hybrids (F1/F2), Backcrossed Saltmarsh Sparrow (BC_SALS), and Saltmarsh Sparrow (SALS) (*denotes significance at the 0.05 confidence level)

Using the observed genotypic distribution of adult birds, a predicted distribution of offspring genotypes was calculated using a contingency table (assuming random mating) for the center and south of the zone and compared to the observed offspring distribution. The predicted distribution of offspring genotypes differed significantly from the observed distributions in both the center (*P* < 0.001, multinomial exact test) and south (*P* < 0.001, multinomial exact test) of the hybrid zone (Fig. 3), suggesting that the resulting genotypic distributions are not directly proportional to those predicted by relative population densities of the genotypic classes. Specifically, the south had a lower proportion of backcrossed Saltmarsh Sparrows (0.54; range CI: 0.49–0.59) than predicted (0.74), and a higher number of pure Saltmarsh Sparrows (0.25; range CI: 0.20–0.31) than expected (0.10), while recent-generation hybrids were similar to expected. The hybrid zone center populations also had a higher proportion of pure Saltmarsh sparrows (0.06; range CI: 0.01–0.12) than predicted (0.00), but a higher proportion of backcrossed Saltmarsh Sparrows (0.56; CI range: 0.51–0.61) than expected (0.35), as well as a lower proportion of recent-generation hybrids (0.12; range CI: 0.07–0.18) than predicted by neutral demographic processes (0.41). Proportions of pure Nelson’s Sparrows and backcrossed Nelson’s Sparrows were similar to expected in both areas of the range (Fig. 3).

We also assessed the influence of neutral demographic processes at a small scale between the two study locations within the center of the hybrid zone. We found a greater number of adult recent-generation hybrids (F1/F2) at the inland site than at the coastal site (t = 2.17, *P* = 0.03). The mean hybrid index and mean interspecific heterozygosity for each site also reflected these patterns. Hybrid index values were higher at the coastal than inland site (t = -4.71, *P* ≤0.001), and sparrows at the inland site showed more mixture between the two species’ gene pools, with higher interspecific heterozygosity at the inland (mean = 0.21 ± 0.02) than coastal site (mean = 0.17 ± 0.15) (t = 2.22, *P* = 0.03; Table 2). There were no pure nestlings sampled from the inland site despite higher levels of pure adults. The observed distribution of offspring genotypes did not differ from that predicted by neutral demographic processes (relative parental genotypic proportions) at the inland site (*P* = 0.83, exact multinomial test), while the distribution of observed offspring did differ from that predicted by demographic processes at the coastal site (P≤0.001, exact multinomial test; Fig. 3), with significantly fewer F1/F2 hybrid and more pure and backcrossed Saltmarsh Sparrows than predicted from random mating based on parental genotype availability, suggesting that neutral demographic process were not the only drivers of introgression at the coastal site, although they did predict the production of pure and back-crossed Nelson’s Sparrow.

#### Exogenous factors

Abundance differed between the inland and coastal hybrid-range-center sites as a function of habitat area. The coastal marsh (~ 15 hectares) is three times larger than the inland site (~ 5 hectares), and the density of adult breeding birds between the sites was similar with 11.1 birds per hectare at coastal site and 10.4 birds per hectare at the inland site. However, we found a large discrepancy in the number of offspring produced at each marsh. The coastal site produced approximately 4 times as many nestlings per marsh area (20.0 birds/ha) than the inland marsh (5.2 birds/ha). Genotypic class proportions differed across the two study sites as expected, with more Nelson and back-crossed Nelson’s adult sparrows on the inland site and more Saltmarsh and back-crossed Saltmarsh adult sparrows on the coastal site (Table 2; Fig. 2). Sparrows at the inland site also had a larger proportion of Nelson’s Sparrow alleles and lower average hybrid index values, while sparrows at the coastal site had more Saltmarsh Sparrow alleles and higher average hybrid index values (t = -4.71, *P* ≤ 0.001; Fig. 1). The distribution of genotypes differed between the two sites (Χ^2^ = 12.2, *P* = 0.002), with significantly more backcrossing towards Nelson’s Sparrow at inland than coastal marshes (t = 2.54, *P* = 0.01). Additionally, overall there were more Saltmarsh Sparrow-like birds at the costal marsh (mean hybrid index = 0.64 ± 0.14) than the inland site (mean hybrid index = 0.43 ± 0.13; Table 2). As noted above, the observed offspring distribution at the inland marsh did not differ from that predicted by parental genotypes, however, such a difference was observed at the coastal marsh among recent generation hybrids, back-crossed Saltmarsh Sparrows, and pure Saltmarsh Sparrows (Fig. 3). An increase in the proportion of Saltmarsh Sparrow individuals and alleles in this second generation in the coastal marsh is consistent with an influence of local adaptation on introgression as Saltmarsh Sparrows have higher fitness in this environment (Fig. 3).

**Fig. 4:**
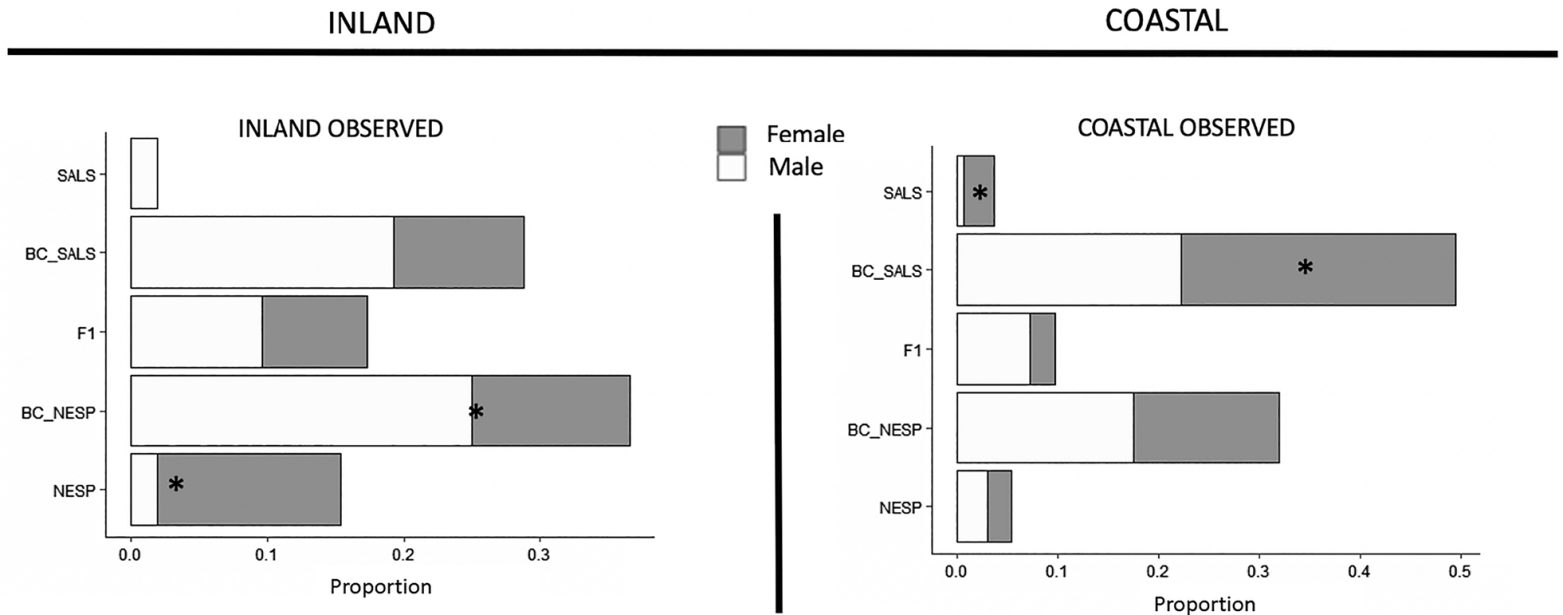
Hypothesis testing for exogenous environmental factors of natural selection. The two panels depict the observed genetic composition of Saltmarsh and Nelson’s Sparrow adult males (white) and females (gray) across the inland (left) and coastal (right) sites (2016 & 2017 seasons). Genotypic classes in all panels: Nelson’s Sparrow (NESP), Backcrossed Nelson’s Sparrow (BC_NESP), F1/F2 (recent-generation hybrids), Backcrossed Saltmarsh Sparrow (BC_SALS), and SALS (Saltmarsh Sparrow) (*denotes significance at the 0.05 confidence level)

The distribution of genotypic classes of females differed between sites (*P* < 0.001, Fisher Exact Test), with more Nelson Sparrow-like adult females at the inland marsh when compared to males, and more Saltmarsh Sparrow-like adult females at coastal marsh when compared to males (Fig. 4). Conversely, there was no difference in the distribution of genotypic classes for adult males (*P* = 0.68, Fisher Exact Test), consistent with our predictions of stronger habitat affinity in females. The inland site had relatively equal proportions of adult F1/F2 individuals by sex with slightly more females (9.6% female F1/F2, 7.7% male F1/F2), while the adult sex ratio of F1/F2 at the coastal site was strongly male biased (2.4% female F1/F2, 7.2% male F1/F2).

#### Endogenous factors

**Fig. 5:**
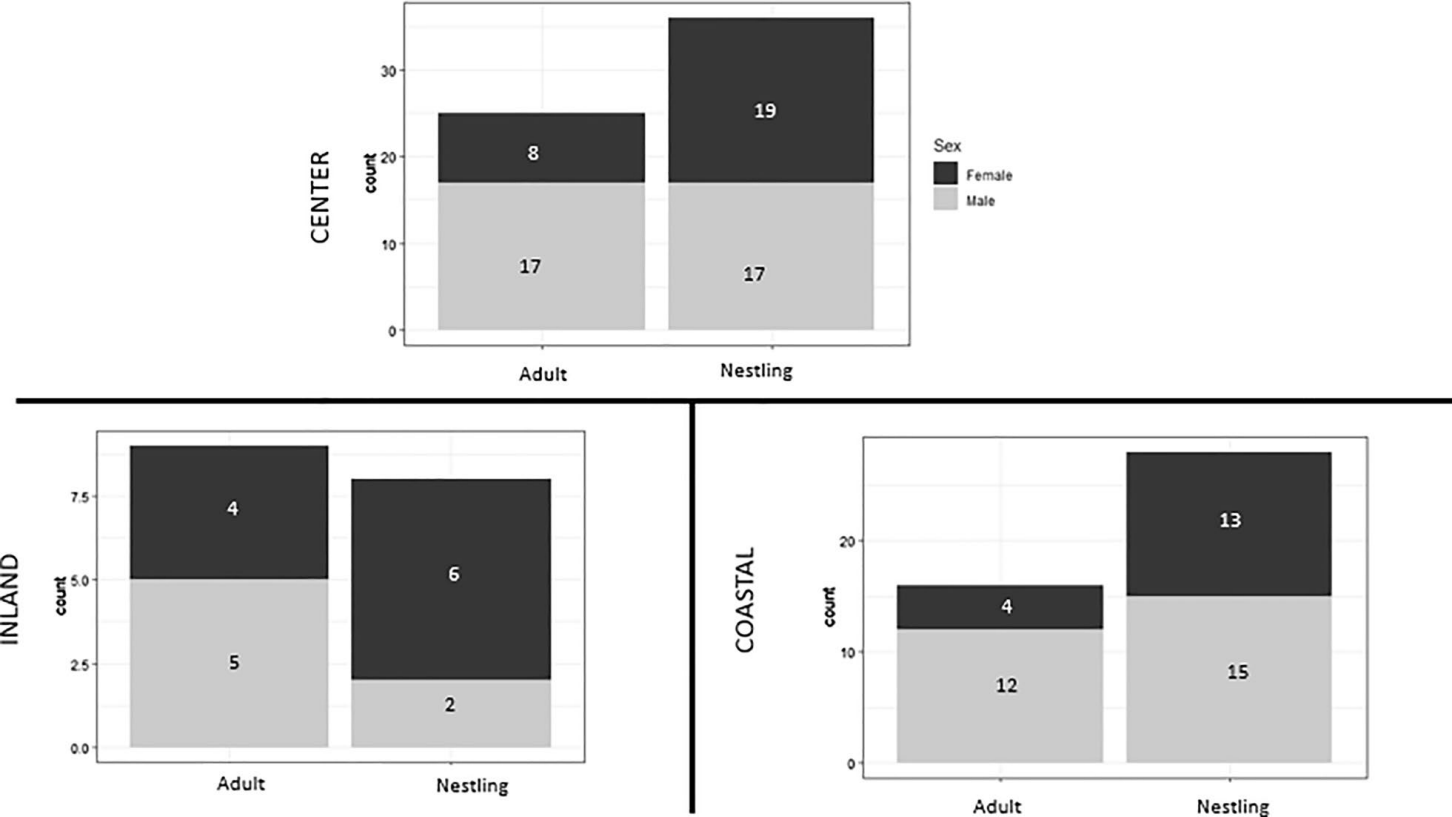
Hypothesis testing for endogenous factors of natural selection. Top panel depicts sex ratio of all recent-generation hybrid sparrows (F1/F2) across both study sites and years in the center of the hybrid zone (2016 & 2017) for the two age classes: adult and nestling. Bottom two panels show the sex ratio of all recent-generation hybrid sparrows between the inland (left) and coastal (right) study sites within the center of the hybrid zone for the two age classes: adult and nestling. Black shading represents females while the gray shading represents males in all panels

We found no difference in mean hybrid index between male and female nestlings (male: 0.66 ± 0.13, female: 0.68 ± 0.12, t = − 0.75, *P* = 0.46) across both study sites and years in the center of the range, suggesting that offspring production and egg viability were not biased in favor of males, and providing no support for reduced viability of females at the egg stage. We did find evidence for reduced survival of females to adulthood, however, through the comparison of the percentage of recent-generation hybrids between nestlings and adults of the two sexes. Proportionally, male and female recent-generation hybrid nestlings represented a similar sector of the combined center-hybrid-range population, with males and females comprising 8.7% and 7.8% of all nestlings, respectively. For the adult age class, however, recent generation hybrid males outnumbered hybrid females 2:1, with the proportion of recent generation hybrid males (5.2% of all adults) twice that of hybrid females (2.5% of all adults; Fig. 5), as might be expected if female hybrids had reduced survival into adulthood or higher rates of emigration from the range center relative to male hybrids. These patterns were also evaluated separately for the coastal and inland site. On the coastal site, there was an equal proportion of recent-generation hybrids nestlings between sex (4.3% of all female offspring, 5.0% male), and three times more male than female recent-generation hybrid adults (males 7.2%, females 2.4%; Fig. 5). On the inland site, many more recent-generation hybrid female nestlings were produced (23%) than males (7.6%). The proportion of recent-generation hybrid males remained the same between age classes (7.6% of all nestlings, 7.7% of all adults), while the proportion of recent-generation hybrid females were reduced by more than half (23% of all nestlings to 9.6% of all adult birds; Fig. 5), suggesting strong reduction in survival of females from nestling stage to adulthood.

#### Sexual selection

**Fig. 6:**
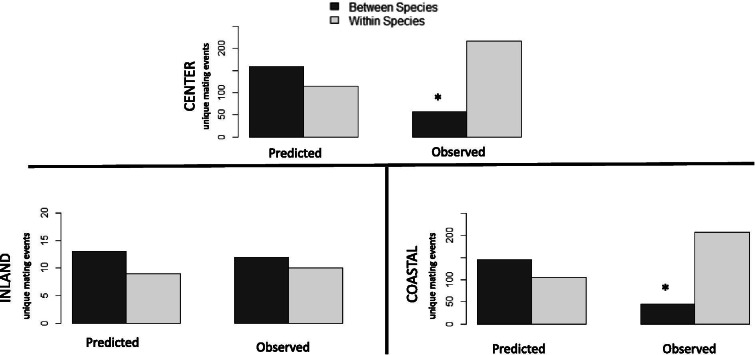
Hypothesis testing for sexual selection. The expected levels of inter (dark gray) and intra (light gray) species mating events based on mate availability compared to the observed levels of between and within-species mating events reconstructed from paternity analyses in the center of the hybrid zone as a whole (top panel), at the inland site (left bottom panel), and at the coastal site (right bottom panel) (*denotes significance at the 0.05 confidence level)

To test for the influence of sexual selection on introgression, we used paternity analyses to reconstruct mating pairs and assigned them to one of two categories: within or between species. The majority (79%) of all reconstructed mating pairs occurred within species groups (when combining backcrossed/pure Saltmarsh Sparrows into a species group and backcrossed/pure Nelson’s Sparrows into another), with 10 times as many matings (217 pairings) within species than between species (21 pairings) across both study sites. The hybrid indices of the parents of each reconstructed mating pair were significantly correlated (r = 0.73, *P* < 0.001), meaning birds were pairing with others that were more like their own genotype. We found this pattern of assortative mating to hold true even when controlling for mate availability, where the proportion of observed between species pairings (34.6%) was much lower than the expected proportion (73.6%), assuming random mating based solely on mate availability (Χ^2^ = 79.5, *P* < 0.001; Fig. 6).

Patterns of mating differed between study sites, with significantly more between species pairings at the inland site (t = 3.30, *P* = 0.003). When controlling for mate availability at each study location, we found that the observed proportion (33.9%) of between species pairings at the coastal marsh was lower than expected (76.4%) by the observed proportions of the genotypic classes (Χ^2^ = 84.3, *P* < 0.001; Fig. 6), indicative of strong assortative mating, while at the inland site there was no difference (Χ^2^ = 0.09, *P* = 0.76; Fig. 6) between the observed (47.4%) proportion of between species mating and that expected by mate availability (52.0%). Further, when we tested for the relationship between mother and father hybrid index of offspring between sites, we found a significant correlation between male and female parental hybrid index scores at the coastal site (r = 0.77, *P* < 0.001), but not at the inland site (r = 0.23, P = 0.30).

## Discussion

Here we sought to disentangle the influence of demographic and selective processes in patterns of introgression in a mosaic hybrid zone between Saltmarsh and Nelson’s Sparrows. We found that neutral demographic factors—relative abundances of the two species—alone could not explain the observed patterns of introgression between Saltmarsh and Nelson’s Sparrows and that spatial variation in the distribution of parental and offspring genotypes was a result of both exogenous and endogenous selective forces. In addition, sexual selection played a role in maintaining species boundaries through assortative mating. However, these patterns differed on the coastal and inland site, suggesting local differences in the strength of selection.

### Natural selection shapes patterns of introgression

Comparing introgression rates in relation to expectations from a neutral demographic model revealed evidence of selective forces influencing hybrid zone structure in this study. Evaluated broadly, neither the center nor the southern edge of the hybrid zone met theoretical predictions of random mating with respect to parental genotype distributions. While high levels of introgression via back-crossing characterized both the southern edge [41] and the center of the Saltmarsh-Nelson’s Sparrow hybrid zone, the direction and asymmetry of introgression differed in a manner largely consistent with relative species distributions and relative abundances. Back-crossing was highly skewed in the direction of the Saltmarsh Sparrow in the south and more equal in both directions in the center, where the two parental species occurred in relatively more similar proportions. However, even in the center, the rate of back-crossing to Saltmarsh Sparrow was greater than predicted by chance, suggesting that both F1/F2 hybrid production and rates of advanced generation back-crossing were influenced by non-neutral factors. Pure and back-crossed Nelson’s Sparrows, on the other hand, occurred with a similar frequency to the expectations of a random-mating model, regardless of their relative abundance or environmental context.

While there were differences in the patterns of advanced introgression via back-crossing across the study area on a broad scale, the production of recent-generation hybrids was remarkably similar (and low)—comprising 12% of each population. With higher access to interspecific mates and relatively equal species abundances in the center of the zone, we expected to find higher rates of hybridization, if population abundance and species distributions were the primary drivers of gene flow. Similar rates of recent-generation hybrids and significantly fewer hybrids in the center of the zone than expected by chance, however, provides evidence that neutral demographic processes alone are insufficient to explain introgression rates and that natural and/or sexual selection may help explain observed patterns of gene flow.

On a fine scale, between the inland and coastal habitats in the center of the zone, we found differences in the relative influence of neutral demographic processes and exogenous selection on introgression. First, consistent with prior work [38] in this mosaic hybrid zone, we found that the genotypic composition of adults differed between the inland and coastal study sites in a manner that supports known differences in habitat affinities and evolutionary histories between the two species. This supports the critical role of habitat preference in fine-scale structure of mosaic hybrid zones [14, 16]. The genotypic distributional differences between the inland and coastal sites were more pronounced in adult females than in adult males, suggesting stronger fitness-related habitat affinities for females, as predicted if exogenous selection were a factor in the structure of this hybrid zone.

Nesting success of females is linked closely with habitat characteristics and nest site selection in this system, with the strongest driver of success being nesting behaviors that mitigate tidal flooding [42–45]. Differential adaptation supports the prevalence of Saltmarsh Sparrow alleles on coastal sites, where Nelson’s Sparrow females have a fitness disadvantage relative to inland marshes, which have a dampened tidal regime [38, 41]. Exogenous selection, therefore, likely plays an important role in shaping introgression patterns in this hybrid zone through differential adaptation and fitness of females. Its relative influence, however, appeared to vary spatially. Offspring genotypic composition on the coastal marsh revealed a deficit of first-generation hybrids and elevated proportions of Saltmarsh and back-crossed Saltmarsh Sparrows, consistent with selection against hybrids and a fitness advantage for birds with Saltmarsh Sparrow alleles in coastal habitats. Conversely, rates of hybridization and introgression were influenced to a greater extent by demographic processes at the inland marsh, suggesting exogenous selection is not as influential, due potentially to different interspecific dynamics on this small marsh or to its local environment. Alternatively, because pure and back-crossed Saltmarsh Sparrows were relatively common on the coastal site and relatively rare on the inland site, these patterns may be explained by processes occurring only in this species.

In addition to exogenous selective factors, we found that endogenous selection also plays a role in structuring this hybrid zone. Specifically, reduced hybrid female survival may be acting as a post-zygotic isolating mechanism, as our results indicate a reduction in survival of female hybrids consistent with Haldane’s Rule, mirroring findings from the southern edge of the hybrid zone [41]. While there was no evidence for selection acting to reduce the production or viability of female eggs, there was a reduction in the proportion of recent-generation hybrid female adults relative to nestlings, in contrast to a relatively similar proportion of recent-generation hybrid male adults and nestlings. This pattern was also evident in the overall sex ratio between adult and nestling F1/F2 hybrids (equal in nestlings—47:53, male biased in adults—68:32).

### Sexual selection maintains species boundaries

Behavior and mate choice are important in determining hybrid zone structure and patterns of introgression, because the occurrence of hybridization is often due to a breakdown of premating isolation [27, 46]. Although variation in behavior across hybrid zones can lead to differing patterns of hybridization and introgression [25, 47], we found that interspecific mate choice was consistent across the Saltmarsh-Nelson’s Sparrow hybrid zone at a broad scale. Similar to trends in the southern part of the zone [48], we observed preference for within-species matings in the center of the hybrid zone, with the large majority of the reconstructed mating events (79%) within species boundaries. Within-species mating preference held true even when accounting for mate availability, further supporting the conclusion that individuals prefer genotypically similar (conspecific) mates.

When inter-specific mating patterns were examined on a fine scale across the study sites in the center of the zone, we found local patterns differed by site. Specifically, assortative mating was strong at the coastal site, but not at the inland site, which showed no difference between the observed level of interspecific mating and that predicted by random mate choice. Thus, the evidence for assortative mating in the center of the zone was driven by the coastal site, where Saltmarsh Sparrows were more abundant. Indeed, only the production of back-crossed and pure Saltmarsh Sparrow offspring differed from that predicted by neutral processes in the coastal population.

Pure and back-crossed Saltmarsh Sparrow offspring were not produced in greater abundance than expected in the inland, hybrid-range-center site, and this could be a result of (A) habitat-specific mating preferences in Saltmarsh Sparrows (i.e., they are more likely to prefer intraspecific mates in the environment where Saltmarsh Sparrow alleles have a fitness advantage), (B) density-specific mating preferences (i.e., they are more likely to express intraspecific mating preferences where Saltmarsh Sparrows are common), or (C) small population size and the relative rarity of pure and back-crossed Saltmarsh Sparrows in the inland site prevented us from detecting intraspecific mating preferences statistically. Regardless, back-crossed and pure Nelson’s Sparrow offspring were produced similarly to neutral expectations in every environmental and every demographic context we examined, including both inland and coastal sites and in hybrid-range-center and southern study locations.

Observed patterns of assortative mating cannot be explained by limited access to conspecific mates, especially in the range-center populations. Rather, some mechanisms(s) of prezygotic reproductive isolation may be acting to limit hybridization and maintain species boundaries. This could take shape in the form of male-male competition for access to mates, sperm competition, female choice (overt or cryptic), or a combination, at either the pre- or post-copulatory stage [49–51]. Mate choice can be based on numerous kinds of male secondary sexual traits or sexual signals that influence pre-copulatory decisions, including traits involved in competition (encounter rates), fighting (body size), and dominance signaling (song, or mate guarding) [50]. Although both Saltmarsh and Nelson’s Sparrows lack sexual dimorphism in plumage, they do exhibit size dimorphism, as well as differences in song and mating behavior that may act as sexual signals. Nelson’s Sparrows are smaller in size, more likely to mate guard, and exhibit flight displays and a louder, albeit simpler, song [35, 44, 52]. These differences may underlie differences in competitive ability or cryptic female choice, such that within species matings are more successful or more likely to occur than those between species.

Differential patterns in assortative mating between sites in the center of the hybrid zone is consistent with the demographic expectation of increased hybridization due to the breakdown of isolating mechanisms in small populations [53]. The inland marsh is much smaller than the coastal, with a smaller population size, which could increase the number of interspecific interactions [11, 54]. It thereby suggests that, like natural selection, sexual selection exerts a stronger influence on coastal relative to inland sites, where rates of introgression are primarily influenced by neutral demographic processes. Alternatively, Saltmarsh Sparrows may have higher intraspecific mate affinity than Nelson’s Sparrows everywhere and observed patterns between coastal and inland sites may be due to a deficit of Saltmarsh Sparrow-like birds and a higher majority of Nelson Sparrow-like birds at inland when compared to coastal locations.

## Conclusion

We found evidence for all hypothesized drivers—neutral demographic processes, exogenous selection, endogenous selection, and sexual selection—influencing variation in patterns of introgression within the Saltmarsh-Nelson’s Sparrow hybrid zone, with site-specific differences in the relative influence among them. Firstly, the overall patterns of introgression on a broad spatial scale could not be explained by neutral demographic processes alone. As is typical of mosaic hybrid zones, local site-specific environmental characteristics shape the distribution of genotypes across the Saltmarsh-Nelson’s Sparrow hybrid zone through differential habitat affinities and exogenous selective pressures. Reduced fitness of females with Nelson’s Sparrow alleles in coastal marshes and reduced survival of hybrid females between the habitats may limit the extent of hybridization, especially on coastal sites, where environmental selective pressures related to nesting ecology are the strongest. In turn, sexual selection further acts to separate the species via interspecific mate avoidance, particularly by Saltmarsh Sparrows on coastal and southern hybrid-range sites where they are most common. Neutral demographic effects appear to dominate on the smaller, inland sites, where Nelson’s Sparrows are more common, bird abundances in general are low, and selection on nest-site preferences are weaker. These findings highlight the context-dependent factors that influence the dynamics and structure of hybrid zones.

## Methods

### Study sites

Two field sites were selected near the center of the hybrid zone: a coastal marsh at Popham Beach State Park and an inland marsh at Wharton Point on Maquoit Bay, located on the northeastern coast of the United States, between Brunswick, Maine and Phippsburg, Maine (Fig. 1). We chose these sites with expectations of relatively similar species abundances based on recent abundance estimates [55] and a relatively high number of first-generation hybrids based on a peak in interspecific heterozygosity across the hybrid zone [40]. Leveraging prior work in the southern end of the Saltmarsh-Nelson’s Sparrow hybrid zone [38], we were able to compare levels of hybridization and patterns of introgression on a large spatial scale between the center and southern range margins of the hybrid zone (~ 100 km from the center); including three previously sampled marsh locations: Chapman’s Landing in Stratham, New Hampshire (latitude 43.041; longitude − 70.924); Lubberland Creek in Newmarket, New Hampshire (latitude 43.073; longitude − 70.903); and Eldridge Marsh in Wells, Maine (latitude 43.292; longitude − 70.572).

The two study locations differ in fine-scale habitat (vegetation) characteristics and amount of tidal inundation [39], allowing us to assess patterns of introgression on a small spatial scale between a coastal and inland tidal marsh (~ 20 km apart). The coastal marsh (15-ha plot) at Popham Beach State Park is located at the tip of a peninsula, behind a sand dune system on the Gulf of Maine. The inland marsh is located 7 km inland from the mouth of Maquoit Bay where it meets Casco Bay and 20 km inland from the Gulf of Maine. It is a much smaller marsh (5-ha plot) than the coastal site. Further, the coastal marsh is part of an expansive coastal marsh network, while the inland marsh is located in a small cove that is surrounded by mostly forest and field. Although both sites experience daily and monthly tidal inundation, tide heights tend to be dampened in inland marshes relative to coastal [45]. In addition, through water-level monitoring on the marsh over the two-year study period, we know flooding rates were lower at the inland site compared to the coastal site [56].

### Field data collection

To determine the extent of hybridization and patterns of introgression, we sampled the population at both sites during the 2016 & 2017 breeding seasons (Additional file 2 describes all sampled individuals). We followed standardized protocols established by the Saltmarsh Habitat and Avian Research Program (SHARP; www.tidalmarshbirds.org). We performed systematic and opportunistic netting, using 2–6 12-m mist-nets throughout the breeding season to sample the resident adult population. To test predictions of Haldane’s rule and assortative mating, we sampled as many offspring as possible. We conducted intensive nest monitoring at both sites during May—August, encompassing approximately 3 nesting cycles (following SHARP nest monitoring protocols; www.tidalmarshbirds.org). From each nest, nestlings were banded with a USGS aluminum leg band and a single site-specific color band when they were 6 days old. A blood sample (a few drops on a filter card) was also collected from the medial metatarsal vein of each nestling for genotyping, hybrid identification, and molecular sex determination. We also collected any deceased, unbanded chicks (most chick death occurred due to flooding, which allows for carcass recovery) or eggs that had failed to hatch to use in genetic analyses. To determine the identity of females associated with each nest, we conducted targeted mist-netting to capture females off of their nests during incubation or brooding. Once caught, each female was banded with a USGS aluminum band, a site-specific color band, and a PIT tag that was attached to a color band for non-invasive detection of re-nesting attempts. Males were sampled systematically and opportunistically across each study site and throughout the breeding season and banded with a USGS aluminum band and a site-specific color band. We collected standard morphological measurements from all adults and recorded presence/absence of brood patch for females. Blood samples were drawn from the cutaneous ulnar vein and stored on blood filter strips at room temperature for genetic analyses.

### ddRAD library preparation

Samples from adult females, nestlings, and salvaged chicks or eggs from the two field seasons were used to prepare double digest restriction site associated DNA (ddRAD) sequencing libraries. In addition, we also used 30 samples each from allopatric Nelson’s Sparrow (Upper Naraguagus, Maine; Wolfville and Yarmouth, Nova Scotia; Hobart Stream, Maine) and allopatric Saltmarsh Sparrow populations (Sawmill Creek, Idlewild, Marine Nature Center and Shirley, New York; Sachuset, Rhode Island; Barn Island, Connecticut) from previous sampling of the hybrid zone for developing a hybrid index (Additional file 1; Fig. 1). DNA was extracted from blood samples using either Qiagen DNeasy Blood and Tissue kit (Qiagen, Valencia, CA) or Zymo Quick DNA kit (Zymo, Irvine, CA) following the manufacturer’s protocol. We determined the concentration of resulting DNA samples using Qubit fluorometer Broad Range double-stranded DNA assay kit (Life Technologies, NY, USA). We targeted a DNA concentration of 5*–*25 ng/ul. Samples below 10 ng/ul after initial extraction were vacuum centrifuged to concentrate to within the target range. Samples that were above 25 ng/ul were diluted down to 25 ng/ul. A small number of samples below 5 ng/ul were included and grouped into one index group to ensure the best results*.* ddRAD libraries were created using the protocol described in Peterson et al. [57, 58]. DNA was digested with SbfI and MspI and ligated to P1 and P2 adapters using T4 DNA ligase (30 min at 37 °C and 60 min at 20 °C, held at 10 °C) [58]. Samples were pooled into index groups by their unique P1 adapter and cleaned using 1.5 × Agencourt AMPure XP beads. Using BluePippin (Sage Science, MA, USA), fragments were size selected between 400–700 bp in length. Low cycle PCR reactions were then performed to incorporate the Illumina TruSeq primer sequences into the library, as well as a final clean up using AMPure XP beads. Libraries were visualized on a fragment Bioanalyzer to ensure desired fragment size/distribution and index groups pooled. Resulting libraries were sequenced across three Illumina HiSeq 2500 lanes and one HiSeq 2500 rapid run lane (read length 100 bp) at the Cornell University Institute for Biotechnology (Genomics Facility Research Center).

### Bioinformatic data processing and SNP detection

Sequences were initially evaluated for overall quality using FastQC, then trimmed and filtered using FASTX-Toolkit. Specifically, reads were trimmed on the 3′ end to 97 bp and eliminated if the Phred quality scores were below 10 or if 95% of the bases had Phred quality scores below 20. Using STACKS (version 1.48), we demultiplexed the remaining sequences. We used the process_radtags command with the following conditions: any reads not meeting Illumina’s chastity/purity filter and of low quality were discarded, data were cleaned such that any read with an uncalled base was removed, reads with mismatches in the adapter sequence > 1 were removed, and reads were only processed if the sequence had an intact SbfI RAD site and one of the unique barcodes. Subsequently, fastx_trimmer was used to trim all sequences to the length of the shortest sequences. Reads were aligned to the Saltmarsh Sparrow reference genome [59] using Bowtie 2 end-to end option (version 2.2.9). STACKS (version 1.48) was used to identify SNPs. Minimum stack depth for a read to be assembled into a catalog was 6. The number of mismatches allowed between sample loci was set at 5. We filtered catalog loci based on the mean log likelihood of the catalog locus in the population, with the minimum log likelihood set at -300. These filtering steps resulted in the recovery of 5,391 SNPs.

We used the program Populations to subset a panel of SNPs for use in calculating a hybrid index. We chose only one SNP per locus and required that a SNP be present in a minimum of 50% of all individuals, with a minimum stack depth of 6, for it to be included. Subsequently, VCFtools [60] was used to group individuals into 3 populations: (1) all individuals sampled in this study from the center of the hybrid zone, (2) allopatric Nelson’s Sparrows, and (3) allopatric Saltmarsh Sparrows. We then calculated the fixation index (F_st_) for each SNP using VCFtools and subsetted the panel further to include only fixed SNPs (F_st_ = 1) between allopatric Nelson’s and Saltmarsh Sparrows. This resulted in a panel of 135 fixed SNPs that we used for the development of a hybrid index to classify pure and hybrid individual sparrows (Additional file 3).

We also created a separate panel of SNPs to be used in paternity analysis to address questions about assortative mating using only sympatric birds from the inland and coastal study sites (i.e., excluding allopatric samples; Additional file 4). For the paternity panel we again chose only one SNP per locus and required that a SNP be present in a minimum of 95% of the individuals with a minimum stack depth of 6. This resulted in a 589-SNP paternity panel.

### Determining genotypic classes

Sparrows were assigned to genotypic classes using methods of Milne and Abbot (2008) [61], as in Walsh et al. [38]. Using this method, which combines hybrid index and interspecific heterozygosity, we placed each individual into one of five genotypic classes consisting of: pure Nelson’s Sparrow, backcrossed Nelson’s, F1/F2 (recent generation hybrids), backcrossed Saltmarsh, or pure Saltmarsh Sparrow. Hybrid index was defined as the proportion of alleles inherited from the Saltmarsh Sparrow (0 = pure Nelson’s Sparrow and 1 = pure Saltmarsh Sparrow), based on the 30 allopatric Saltmarsh and Nelson’s sparrows. Interspecific heterozygosity was defined as the proportion of genotypes that were heterozygous across the species for the parental alleles (0 = all homozygous genotypes, found only in one parental species, and 1 = all heterozygous genotypes across species). Individuals with intermediate hybrid index (0.25–0.75) and high heterozygosity (> 0.3) were considered recent generation hybrids (F1 or F2), and individuals with very low or high hybrid index (0.05–0.25 or 0.75–0.95) and low heterozygosity (< 0.3) were considered backcrossed. Pure individuals were defined by a hybrid index of 0–0.05 (Nelson’s Sparrow) or 0.95–1 (Saltmarsh Sparrow). The Introgress package in R was used for calculating the hybrid index and interspecific heterozygosity [62]. Analyses did not distinguish between F1 and F2 individuals, which were grouped together into an overall recent-generation hybrid category, used throughout. Genetic composition of the coastal and inland populations were compared to allopatric parental populations (Saltmarsh and Nelson’s) using structure, version 2.3.4 [63] and visualized using CLUMPAK [64].

### Paternity analyses

We conducted paternity analyses of nestlings using genotype data from the SNP paternity panel and reconstructed mating pairs. Candidate fathers were assigned using the approaches implemented in cervus [65] and colony v2.0 [66]. The maximum likelihood approach of CERVUS uses simulated genotypes from provided data to create a log-likelihood confidence level in true parentage assignments but does not account for unsampled males in the population. To address this problem, we used the full likelihood approach in COLONY, which can assign paternity to a sampled male even if the true father was not among the sampled males. For both methods, we used a genotyping error rate of 1, 95% of loci typed, and candidate father sampling of 70%. We assumed the proportion of sampled mothers to be 95% given the targeted netting identification of females off of their nests. For each site and year, a list of candidate fathers was developed. For 2016, all sampled adult males were included, and for 2017, all males that were sampled in that year, as well as any males from 2016 (adults and offspring as determined from molecular sexing) were included to account for any hatch years that may have returned to their natal site, as well as any returning adult males that may have evaded capture in 2017. For each offspring, we determined the most likely father as assigned by CERVUS (delta trio value ≥ 95%). This was then compared to the paternity assignment made in COLONY. For any discrepancies on confident paternity assignments (> 95%) between the two programs, we compared the number of loci mismatches, delta pair confidence, and overall loci typed to identify the best male assignment. The sex of each offspring was identified by PCR amplification of the CDH1 gene [67, 68] and visualized using gel electrophoresis.

### Hypothesis testing

#### Demographic processes

To test for the influence of relative population density on patterns of introgression, we compared the distribution of genotypes for all individuals (nestlings and adults) between the center and the south of the hybrid zone and between inland and coastal sites within the center of the hybrid zone. Using the observed genotypic distribution of adult birds, a predicted distribution of offspring genotypes was calculated using a contingency table, assuming random mating dependent on the relative abundance of each observed genotypic class for the center and south of the zone, as well as for each site within the center of the hybrid zone, separately. In this contingency table, pure Nelson’s mating with pure Nelson’s Sparrow resulted in another pure Nelson’s Sparrow; and similarly, pure Saltmarsh Sparrow mating with pure Saltmarsh Sparrow also produced a pure species designation. Backcrossed Nelson’s Sparrows within the contingency table were produced from the following three crosses: backcrossed Nelson’s with pure Nelson’s, backcrossed Nelson’s with F1/F2, and backcrossed Nelson’s with backcrossed Nelson’s. Similarly, backcrossed Saltmarsh Sparrows were the result of any of the following three crosses: backcrossed Saltmarsh with pure Saltmarsh, backcrossed Saltmarsh with F1/F2, and backcrossed Saltmarsh with backcrossed Saltmarsh. A mating between a Saltmarsh and Nelson’s Sparrow, a backcrossed Saltmarsh Sparrow and backcrossed Nelson’s Sparrow, as well as a pure Nelson’s with a backcrossed Saltmarsh, or a pure Saltmarsh and a backcrossed Nelson’s were considered F1/F2 designation. Subsequently the observed offspring distribution for each site and hybrid zone location were compared to the predicted offspring genotypic composition using a Goodness of fit Exact Multinomial Test with Monte Carlo approach (ntrial = 100,000) using the EMT package in R (Version 1.1). If patterns of gene flow were controlled by neutral demographic processes alone, we would expect that the observed offspring distribution would be proportional to the one predicted assuming random mating dependent on observed relative population densities of each genotypic class. To determine if there were higher levels of hybridization at the inland or coastal site, we compared the number of recent-generation hybrids (F1/F2 class) between the coastal and inland site using a two-tailed Student’s *T*-test. We also compared the mean interspecific heterozygosity and hybrid index scores between the study site locations.

#### Exogenous environmental factors

To test for the influence of habitat on patterns of introgression, we compared the distribution of the genotypic classes between coastal and inland site using a chi-squared test. In addition, two-tailed Student’s t-tests were performed to compare the proportion of backcrossed individuals, mean hybrid index, and mean interspecific heterozygosity levels between the two sites to determine if there was more backcrossing towards Nelson’s Sparrow at inland site and more backcrossing towards Saltmarsh Sparrow at the coastal, as predicted based on recorded directions of local adaptation from previous study [38]. Finally, we compared the observed offspring genotypic class at each site to the predicted distribution based on demographic processes (described above). If habitat were acting on patterns of gene flow, we could expect that the observed offspring genotypic class would differ from that predicted by demographic processes alone, and, specifically, that the backcrossed Nelson’s and pure Nelson’s categories would be higher than expected by random mating at the inland site and the backcrossed Saltmarsh and pure Saltmarsh categories would be higher than expected randomly at the coastal site. The observed offspring distribution for each site was compared to the predicted offspring genotypic composition using a Goodness of fit Exact Multinomial Test with a Monte Carlo approach (ntrial = 100,000). Multinomial confidence intervals were calculated for a post-hoc test to determine which categories differed, such that estimates with confidence intervals that did not contain the theoretical proportion were identified as different from the predicted.

Additionally, if habitat were influencing patterns of introgression, we would expect nesting females to be more site selective than males as a result of the fitness consequences of settlement patterns. We predict that there will be more Nelson-like females nesting at the inland marsh and more Saltmarsh-like females nesting at the coastal marsh, due to known relationships between habitat and nesting success for the two species [41]. To test this, we compared the female genotypic distribution between sites using a Fisher Exact Test for count data. To compare this to observed patterns for males, we then tested the distribution of male genotypic classes between sites, also using a Fisher Exact Test for count data.

#### Endogenous factors

To test Haldane’s Rule about the sex-biased effects of genetic incompatibilities in hybrids, we determined: (1) if production of recent-generation hybrids was male-biased due to offspring sex ratio manipulation or reduced viability of female eggs or offspring; or (2) if there was reduced occurrence of hybrid females from the nestling to adult stage, suggesting a reduction in survival. To assess reduced survival of females, we compared the proportion of recent generation hybrids among nestling females, adult females, nestling males, and adult males using a 2-sample test for equality of proportions. We performed two-tailed Student’s t-tests to compare the hybrid index of male and female offspring across both sites and the proportion of male offspring produced from interspecific and intraspecific mating events.

#### Sexual selection

To test for the influence of sexual selection on patterns of introgression, we sought evidence of assortative mating using the results of the paternity analyses. Each mating event was classified into two categories: within or between species. The within species mating included: Nelson’s Sparrow with Nelson’s Sparrow (including backcrossed), and Saltmarsh Sparrow with Saltmarsh Sparrow (including backcrossed). Between species category included: F1/F2 with Nelson’s Sparrow (pure or backcrossed), F1/F2 with Saltmarsh Sparrow (pure or backcrossed), and Nelson’s Sparrows (pure or backcrossed) with Saltmarsh Sparrow (pure or backcrossed). The number of unique mating events resulting from each group was compared. To account for mate availability, an expected distribution of between and within species pairings was determined based upon observed frequencies of the genotypic classes. The proportion of observed between and within species mating pairs was compared to the expected distribution using a 2-sample test for equality of proportions. Under assortative mating, we would expect the observed proportion of between species pairings would be lower than expected levels of interspecies mating assuming random mating.

Assortative mating was also compared between coastal and inland study sites. An expected proportion of within and between species matings were determined for both coastal and inland marshes and compared to observed levels of inter and intra species pairings using a 2-sample test for equality of proportions. Additionally, we compared mating patterns between coastal and inland, testing for differences in the proportion of between species and within species mating across the two sites using a two-tailed Student’s t-test. Finally, we tested for a correlation between the parental hybrid index scores for each offspring using a Pearson product-moment correlation coefficient across all individuals and between our two hybrid-range-center study sites.

## Supplementary Information

**Additional file 1.** List of Allopatric Saltmarsh and Nelson's Sparrow individuals used for hybrid index.

**Additional file 2.** Metadata for sparrow individuals from 2016 & 2017 sampled in this study .

**Additional file 3.** Fixed SNP panel (135) for all individuals.

**Additional file 4.** Paternity SNP panel (589) for hybrid zone center individuals.

## Data Availability

All data generated or analyzed during this study are included in this published article and its supplementary information files.

## Abbreviations

SNP: Single nucleotide polymorphism
PIT tag: Passive Integrated Transponder
USGS: United States Geological Survey
ddRAD: Double digest Restriction-site Associated DNA
PCR: Polymerase chain reaction
SALS: Saltmarsh Sparrow
NESP: Nelson’s Sparrow
BC_SALS: Backcrossed Saltmarsh Sparrow
BC_NESP: Backcrossed Nelson’s Sparrow

## References

[1] Ross CL, Harrison RG (2002). A fine-scale spatial analysis of the mosaic hybrid zone between gryllus firmus and gryllus pennsylvanicus. Evolution.

[2] Morgan-Richards M, Wallis GP (2003). A comparison of five hybrid zones of the weta hemideina thoracica (orthoptera: anostostomatidae): degree of cytogenetic differentiation fails to predict zone width. Evolution.

[3] Harrison RG, Larson EL (2014). Hybridization, Introgression, and the Nature of Species Boundaries. J Hered.

[4] Futuyma DJ, Shapiro LH (1995). Hybrid zones. Evolution.

[5] Vines TH, Kohler SC, Thiel M, Ghira I, Sands TR, MacCallum CJ (2003). The maintenance of reproductive isolation in a mosaic hybrid zone between the fire-bellied toads *bombina bombina* and *b. variegata*. Evolution.

[6] Burgess KS, Morgan M, Deverno L, Husband BC (2005). Asymmetrical introgression between two Morus species (*M. alba*, *M. rubra*) that differ in abundance. Mol Ecol..

[7] Dabrowski A, Fraser R, Confer JL, Lovette IJ (2005). Geographic variability in mitochondrial introgression among hybridizing populations of Golden-winged (*Vermivora chrysoptera*) and Blue-winged (*V. pinus*) Warblers. Conserv Genet..

[8] Field DL, Ayre DJ, Whelan RJ, Young AG (2010). Patterns of hybridization and asymmetrical gene flow in hybrid zones of the rare *Eucalyptus aggregata* and common *E. rubida*. Heredity.

[9] Hubbs CL (1955). Hybridisation between fish species in nature. Syst Zool.

[10] Randler C (2002). Avian hybridization, mixed pairing and female choice. Anim Behav.

[11] Ellstrand NC, Elam DR (1993). Population genetic consequences of small population size: Implications for Plant Conservation. Annu Rev Ecol Syst.

[12] Baskett ML, Gomulkiewicz R (2011). Introgressive hybridization as a mechanism for species rescue. Theor Ecol.

[13] Carling MD, Thomassen HA (2012). The role of environmental heterogeneity in maintaining reproductive isolation between hybridizing passerina (Aves: Cardinalidae ) buntings. Int J Ecol.

[14] Culumber ZW, Shepard DB, Coleman SW, Rosenthal GG, Tobler M (2012). Physiological adaptation along environmental gradients and replicated hybrid zone structure in swordtails (*Teleostei: Xiphophorus*). J Evol Biol.

[15] Dubay SG, Whitt CC (2014). Differential high-altitude adaptation and restricted gene flow across a mid-elevation hybrid zone in Andean tit-tyrant flycatchers. Mol Ecol.

[16] Carson EW, Tobler M, Minckley WL, Ainsworth RJ, Dowling TE (2012). Relationships between spatio-temporal environmental and genetic variation reveal an important influence of exogenous selection in a pupfish hybrid zone. Mol Ecol.

[17] Aldridge G, Campbell DR (2009). Genetic and morphological patterns show variation in frequency of hybrids between Ipomopsis (Polemoniaceae ) zones of sympatry. Heredity.

[18] Aldridge G (2005). Variation in frequency of hybrids and spatial structure among Ipomopsis (Polemoniaceae ) contact sites. New Phytol.

[19] Bronson CL, Grubb TC, Braun MJ (2003). A test of the endogenous and exogenous selection hypothesis for the maintenance of a narrow avian hybrid zone. Evolution.

[20] Svedin N, Wiley C, Veen T, Gustafsson L, Qvarnstrom A (2008). Natural and sexual selection against hybrid flycatchers. Proc R Soc B.

[21] Steeves TE, Maloney RF, Hale ML, Tylianakis JM, Gemmell NJ (2010). Genetic analyses reveal hybridization but no hybrid swarm in one of the world’s rarest birds. Mol Ecol.

[22] Barton NH, Hewitt GM (1985). Analysis of hybrid zones. Ann Rev Ecol Syst.

[23] Haldane JBS (1922). Sex ratio and unisexual sterility in hybrid animals. J Genet.

[24] Neubauer G, Nowicki P, Zagalska-Neubauer M (2014). Haldane’s rule revisited: Do hybrid females have a shorter lifespan? Survival of hybrids in a recent contact zone between two large gull species. J Evol Biol.

[25] Pearson SF, Rohwer S (2000). Asymmetries in male aggression across an avian hybrid zone. Behav Ecol.

[26] Pearson SF (2000). Behavioral asymmetries in a moving hybrid zone. Behav Ecol.

[27] Culumber ZW, Ochoa OM, Rosenthal GG (2014). Assortative mating and the maintenance of population structure in a natural hybrid zone. Am Nat.

[28] Nocera JJ, Fitzgerald TM, Hanson AR, Milton GR (2007). Differential habitat use by Acadian Nelson’s Sharp-tailed Sparrows: implications for regional conservation. J Field Ornithol.

[29] Greenlaw J, Woolfenden GE (2007). Wintering distributions and migration of Saltmarsh and Nelson’s Sharp-tailed sparrows. Wilson J Ornithol.

[30] Rising JD, Avise JC (1993). Application of genealogical-concordance principles to the taxonomy and evolutionary history of the sharp-tailed sparrow (*Ammodramus caudacutus*). Ornithology.

[31] Hodgman TP, Shriver WG, Vickery PD (2002). Redefining range overlap between the sharp-tailed sparrows of coastal New England. Wilson Bull.

[32] Shriver WG, Gibbs JP, Vickery PD, Gibbs HL, Hodgman TP, Jones PT (2005). Concordance between morphological and molecular markers in assessing hybridization between Sharp-tailed Sparrows in New England. Auk.

[33] Walsh J, Kovach AI, Lane OP, O’Brien KM, Babbitt KJ (2011). Genetic barcode RFLP analysis of the Nelson’s and Saltmarsh sparrow hybrid zone. Wilson J Ornithol.

[34] Walsh J, Shriver WG, Olsen BJ, O’Brien KM, Kovach AI (2015). Relationship of phenotypic variation and genetic admixture in the Saltmarsh–Nelson’s sparrow hybrid zone. Auk.

[35] Greenlaw JS (1993). Behavioral and morphological diversification in sharp-tailed sparrows (*Ammodramus caudacutus*) of the Atlantic Coast. Auk.

[36] Walsh J, Clucas G, MacManes M, Thomas K, Kovach A (2018). Divergent selection and drift shape the genomes of two avian sister species spanning a saline-freshwater ecotone. BioRxiv..

[37] Walsh J, Kovach AI, Olsen BJ, Shriver WG, Lovette IJ (2018). Bidirectional adaptive introgression between two ecologically divergent sparrow species: Hybridization in Saltmarsh and Nelson’s sparrows. Evolution.

[38] Walsh J, Rowe RJ, Olsen BJ, Shriver WG, Kovach AI (2015). Genotype-environment associations support a mosaic hybrid zone between two tidal marsh birds. Ecol Evol.

[39] Walsh J, Shriver WG, Olsen BJ, O’Brien KM, Kovach AI (2015). Relationship of phenotypic variation and genetic admixture in the Saltmarsh-Nelson’s sparrow hybrid zone. Auk.

[40] Walsh J, Shriver WG, Olsen BJ, Kovach AI (2016). Differential introgression and the maintenance of species boundaries in an advanced generation avian hybrid zone. BMC Evol Biol.

[41] Walsh J, Olsen BJ, Ruskin KJ, Gregory W, Brien KMO, Kovach AI (2016). Extrinsic and intrinsic factors influence fitness in an avian hybrid zone. Biol J.

[42] Gjerdrum C, Elphick CS, Rubega M (2005). Nest site selection and nesting success in Saltmarsh breeding sparrows: the importance of nest habitat, timing, and study site differences. Condor.

[43] Greenlaw JS, Rising JD. Sharp-tailed Sparrow (*Ammodramus caudacutus*). In Poole A, Gills F (Eds.) The Brids of North America, vol. 112. Academy of Natural Sciences. Philadelphia, PA: American Ornithologists’ Union, Washington, DC; 1994.

[44] Shriver WG, Vickery PD, Hodgman TP, Gibbs JP (2007). Flood tides affect breeding ecology of two sympatric sharp-tailed sparrows. Auk.

[45] Benvenuti B, Walsh J, O’Brien KM, Kovach AI (2018). Plasticity in nesting adaptions of a tidal marsh endemic. Ecol Evol..

[46] Taylor EB, Boughman JW, Groenenboom M, Sniatynski M, Schluter D, Gow JL (2006). Speciation in reverse : morphological and genetic evidence of the collapse of a three-spined stickleback (*Gasterosteus aculeatus* ) species pair. Mol Ecol.

[47] Uy JAC, Stein AC (2007). Variable visual habitats may influence the spread of colourful plumage across an avian hybrid zone. J Evol Biol.

[48] Walsh J, Maxwell LM, Kovach AI (2018). The role of divergent mating strategies, reproductive success, and compatibility in maintaining the Saltmarsh—Nelson’s sparrow hybrid zone. Auk Ornithol Adv.

[49] Parker GA (1970). Sperm competition and its evolutionary con-sequences in the insects. Biol Rev.

[50] Andersson M (1994). Sexual selection.

[51] Birkhead TR (1998). Cryptic female choice: criteria for establishing female sperm choice. Evolution.

[52] Hill CE, Gjerdrum C, Elphick CS (2010). Extreme levels of multiple mating characterize the mating system of the Saltmarsh Sparrow (*Ammodramus caudacutus*). Auk.

[53] Selander RK (1971). Systematics and speciation in birds. Avian Biol.

[54] Beysard M, Perrin N, Jaarola M, Heckel G, Vogel P (2012). Asymmetric and differential gene introgression at a contact zone between two highly divergent lineages of field voles (*Microtus agrestis*). J Evol Biol.

[55] Wiest WA, Correll MD, Olsen BJ, Elphick CS, Hodgman TP, Curson DR (2016). Population estimates for tidal marsh birds of high conservation concern in the northeastern USA from a design-based survey. Condor.

[56] Maxwell, LM. Drivers of introgression and fitness in the Saltmarsh-Nelson’s sparrow hybrid zone. 2018. Master’s Thesis. University of New Hampshire, Durham.

[57] Peterson BK, Weber JN, Kay EH, Fisher HS, Hoekstra HE (2012). Double digest RADseq: An inexpensive method for de novo SNP discovery and genotyping in model and non-model species. PLoS ONE.

[58] Thrasher DJ, Butcher BG, Campagna L, Webster MS, Lovette IJ (2018). Double-digest RAD sequencing outperforms microsatellite loci at assigning paternity and estimating relatedness: a prood of concept in a highly promiscuous bird. Mol Ecol Res.

[59] Walsh J, Clucas GV, MacManes MD, Thomas WK, Kovach AI (2019). Divergent selection and drift shape the genomes of two avian sister species spanning a saline-freshwater gradient. Ecol Evol..

[60] Danecek P, Auton A, Abecasis G, Albers CA, Banks E, Depristo MA (2018). The variant call format and VCFtools. Bioinformatics.

[61] Milne RI, Abbott FJ (2008). Reproductive isolation among two infertile *Rhododendron* species: Low frequency of post-F1 genotypes in alpine hybrid zones. Mol Ecol.

[62] Gompert Z, Alex Buerkle C (2010). Introgress: a software package for mapping components of isolation in hybrids. Mol Ecol Res.

[63] Pritchard JK, Stephens M, Donnelly P (2000). Inference of population structure using multilocus genotype data. Genetics.

[64] Kopelman NM, Mayzel J, Jakobsson M, Rosenberg N, Mayrose I (2015). CLUMPAK: a program for identifying clustering modes and packaging population structure inferences across K. Mol Ecol Res.

[65] Marshell TC, Slate J, Kruuk LEB, Pemberton JM (1998). Statistical confidence for likelihood-based paternity inference in natural populations. Mol Ecol.

[66] Jones O, Wang J (2010). COLONY: a program for parentage and sibship inference from multilocus genotype data. Mol Ecol Res.

[67] Fridolfsson AK, Ellegren H (1999). A simple and universal method for molecular sexing of non-ratite birds. J Avian Biol.

[68] Griffiths R, Daan S, Dijkstra C (1996). Sex identification in birds using two CHD genes. Proc R Soc Lond B..

