# Supplementary material for: Patterns of introgression vary within an avian hybrid zone
Source: BMC Ecol Evol. 2021 Jan 28;21:14. doi: 10.1186/s12862-021-01749-1 (PMC7853311; doi:10.1186/s12862-021-01749-1)
Supplement: Supplementary file 1 — Additional file 1. List of Allopatric Saltmarsh and Nelson's Sparrow individuals used for hybrid index. [file 12862_2021_1749_MOESM1_ESM.pdf]

Sample	Site Name	Location	Coordinates		Year	Collector	Reference
198_nesp	Hobart Stream	ME	-67.204266	44.889845	2015	M. Conway	M. Conway Dissertation [1]
201_nesp	Hobart Stream	ME	-67.204266	44.889845	2015	M. Conway	M. Conway Dissertation [1]
205_nesp	Hobart Stream	ME	-67.204266	44.889845	2015	M. Conway	M. Conway Dissertation [1]
300_nesp	Hobart Stream	ME	-67.204266	44.889845	2015	M. Conway	M. Conway Dissertation [1]
8200_nesp	Hobart Stream	ME	-67.204266	44.889845	2015	M. Conway	M. Conway Dissertation [1]
SALS_1241-89105	Sawmill Creek	NY	-74.191103	40.610062	2012	A. Kocek	This Study
SALS_1241-89167	Sawmill Creek	NY	-74.191103	40.610062	2012	A. Kocek	This Study
SALS_1241-89173	Idlewild	NY	-73.745247	40.648212	2012	A. Kocek	This Study
SALS_1361-54712	Idlewild	NY	-73.745247	40.648212	2014	A. Kocek	This Study
SALS_1361-54720	Idlewild	NY	-73.745247	40.648212	2014	A. Kocek	This Study
SALS_1361-54733	Idlewild	NY	-73.745247	40.648212	2014	A. Kocek	This Study
SALS_1361-54759	Marine Nature Center	NY	-73.622075	40.621339	2014	A. Kocek	This Study
SALS_1361-54856	Marine Nature Center	NY	-73.622075	40.621339	2014	A. Kocek	This Study
SALS_1601-37004	Shirly	NY	-72.893452	40.769611	2007	J. Walsh	Walsh et al. 2012 [2]
SALS_1601-37007	Shirly	NY	-72.893452	40.769611	2007	J. Walsh	Walsh et al. 2012 [2]
SALS_2281-67180	Shirly	NY	-72.893452	40.769611	2007	J. Walsh	Walsh et al. 2012 [2]
SALS_2281-67191	Shirly	NY	-72.893452	40.769611	2007	J. Walsh	Walsh et al. 2012 [2]
SALS_2281-67197	Shirly	NY	-72.893452	40.769611	2007	J. Walsh	Walsh et al. 2012 [2]
SALS_2511-17270	Sachuset	RI	-71.247571	41.486677	2011	E. King	This Study
SALS_2511-17272	Sachuset	RI	-71.247571	41.486677	2011	E. King	This Study
SALS_2511-17274	Sachuset	RI	-71.247571	41.486677	2011	E. King	This Study
SALS_2511-17279	Sachuset	RI	-71.247571	41.486677	2011	E. King	This Study
SALS_2511-17347	Sachuset	RI	-71.247571	41.486677	2011	E. King	This Study
SALS_2541-59170	Barn Island	CT	-71.8627481	41.337466	2014	C. Field/A. Borowske	This Study
SALS_2571-82303	Barn Island	CT	-71.8627481	41.337466	2014	C. Field/A. Borowske	This Study
SALS_2581-97070	Barn Island	CT	-71.8627481	41.337466	2014	C. Field/A. Borowske	This Study
SALS_2581-97281	Barn Island	CT	-71.8627481	41.337466	2014	C. Field/A. Borowske	This Study
SALS_2661-46831	Idlewild	NY	-73.745247	40.648212	2013	A. Kocek	This Study
SALS_2661-46912	Marine Nature Center	NY	-73.622075	40.621339	2014	A. Kocek	This Study
SALS_2661-46932	Sawmill Creek	NY	-74.191103	40.610062	2014	A. Kocek	This Study
SALS_2661-46933	Sawmill Creek	NY	-74.191103	40.610062	2014	A. Kocek	This Study
SALS_2661-46952	Marine Nature Center	NY	-73.622075	40.621339	2014	A. Kocek	This Study
SALS_2661-46953	Marine Nature Center	NY	-73.622075	40.621339	2014	A. Kocek	This Study
SALS_2661-46986	Sawmill Creek	NY	-74.191103	40.610062	2014	A. Kocek	This Study
SALS_2691-08432	Barn Island	CT	-71.8627481	41.337466	2014	C. Field/A. Borowske	This Study

Sample	Site Name	Location	Coordinates		Year	Collector	Reference

upnarr_221_nesp	Upper Narraguagus	ME	-67.913004	44.570516	2015	M. Conway	M. Conway Dissertation [1]
upnarr_222_nesp	Upper Narraguagus	ME	-67.913004	44.570516	2015	M. Conway	M. Conway Dissertation [1]
upnarr_224_nesp	Upper Narraguagus	ME	-67.913004	44.570516	2015	M. Conway	M. Conway Dissertation [1]
upnarr_225_nesp	Upper Narraguagus	ME	-67.913004	44.570516	2015	M. Conway	M. Conway Dissertation [1]
upnarr_227_nesp	Upper Narraguagus	ME	-67.913004	44.570516	2015	M. Conway	M. Conway Dissertation [1]
upnarr_228_nesp	Upper Narraguagus	ME	-67.913004	44.570516	2015	M. Conway	M. Conway Dissertation [1]
wolf_764_nesp	Wolfville	Nova Scotia	-64.213541	45.53034	2015	Walsh/ Kovach	Walsh et al. 2018 [3]
wolf_765_nesp	Wolfville	Nova Scotia	-64.213541	45.53034	2015	Walsh/ Kovach	Walsh et al. 2018 [3]
wolf_766_nesp	Wolfville	Nova Scotia	-64.213541	45.53034	2015	Walsh/ Kovach	Walsh et al. 2018 [3]
wolf_767_nesp	Wolfville	Nova Scotia	-64.213541	45.53034	2015	Walsh/ Kovach	Walsh et al. 2018 [3]
wolf_768_nesp	Wolfville	Nova Scotia	-64.213541	45.53034	2015	Walsh/ Kovach	Walsh et al. 2018 [3]
wolf_769_nesp	Wolfville	Nova Scotia	-64.213541	45.53034	2015	Walsh/ Kovach	Walsh et al. 2018 [3]
wolf_770_nesp	Wolfville	Nova Scotia	-64.213541	45.53034	2015	Walsh/ Kovach	Walsh et al. 2018 [3]
wolf_771_nesp	Wolfville	Nova Scotia	-64.213541	45.53034	2015	Walsh/ Kovach	Walsh et al. 2018 [3]
wolf_772_nesp	Wolfville	Nova Scotia	-64.213541	45.53034	2015	Walsh/ Kovach	Walsh et al. 2018 [3]
yarm_748_nesp	Yarmouth	Nova Scotia	-66.7258	43.501485	2015	Walsh/ Kovach	Walsh et al. 2018 [3]
yarm_749_nesp	Yarmouth	Nova Scotia	-66.7258	43.501485	2015	Walsh/ Kovach	Walsh et al. 2018 [3]
yarm_750_nesp	Yarmouth	Nova Scotia	-66.7258	43.501485	2015	Walsh/ Kovach	Walsh et al. 2018 [3]
yarm_752_nesp	Yarmouth	Nova Scotia	-66.7258	43.501485	2015	Walsh/ Kovach	Walsh et al. 2018 [3]
yarm_753_nesp	Yarmouth	Nova Scotia	-66.7258	43.501485	2015	Walsh/ Kovach	Walsh et al. 2018 [3]
yarm_754_nesp	Yarmouth	Nova Scotia	-66.7258	43.501485	2015	Walsh/ Kovach	Walsh et al. 2018 [3]
yarm_756_nesp	Yarmouth	Nova Scotia	-66.7258	43.501485	2015	Walsh/ Kovach	Walsh et al. 2018 [3]
yarm_759_nesp	Yarmouth	Nova Scotia	-66.7258	43.501485	2015	Walsh/ Kovach	Walsh et al. 2018 [3]
yarm_763_nesp	Yarmouth	Nova Scotia	-66.7258	43.501485	2015	Walsh/ Kovach	Walsh et al. 2018 [3]
yarm_Y1_nesp	Yarmouth	Nova Scotia	-66.7258	43.501485	2015	Walsh/ Kovach	Walsh et al. 2018 [3]

1. Conway, M. **Niche evolution along a gradient of ecological specialization**. 2019. Electronic theses and dissertations. 3025. https://digitalcommons.library.umaine.edu/etd/3025.

2. Walsh, J, Kovach, AI, Babbitt, KJ, O'Brien, KM. **Fine-Scale population structure and asymmetrical dispersal in an obligate Salt-Marsh passerine, the Saltmarsh Sparrow (*Ammodramus Caudacutus*)**. *The Auk*. 2012; 129(2): 247-58.

3. Walsh, J, Kovach, AI, Olsen, BJ, Shriver, WG, Lovette, IJ. **Bidirectional adaptive introgression between two ecologically divergent sparrow species: Hybridization in Saltmarsh and Nelson's sparrows**. *Evolution*. 2018; **72**:2076-2089.
